# Advancements in Additive Manufacturing of Tantalum via the Laser Powder Bed Fusion (PBF-LB/M): A Comprehensive Review

**DOI:** 10.3390/ma16196419

**Published:** 2023-09-27

**Authors:** Aziz Ul Hassan Mohsan, Dongbin Wei

**Affiliations:** School of Mechanical and Mechatronic Engineering, University of Technology Sydney, Sydney, NSW 2007, Australia; azizulhassan.mohsan@student.uts.edu.au

**Keywords:** additive manufacturing, Laser Powder Bed Fusion (PBF-LB/M), tantalum, mechanical properties

## Abstract

Additive manufacturing (AM) exhibits a prime increment in manufacturing technology development. The last few decades have witnessed massive improvement in this field of research, including the growth in the process, equipment, and materials. Irrespective of compelling technological advancements, technical challenges provoke the application and development of these technologies. Metal additive manufacturing is considered a prime sector of the industrial revolution. Various metal AM techniques, including Selective Laser Sintering (SLS), Laser Powder Bed Fusion (PBF-LB/M), and Electron Beam Powder Bed Fusion (PBF-EB/M), have been developed according to materials and process classifications. PBF-LB/M is considered one of the most suitable choices for metallic materials. PBF-LB/M of tantalum has become a hot topic of research in the current century owing to the high biocompatibility of tantalum and its high-end safety applications. PBF-LB/M of porous Ta can direct unexplored research prospects in biomedical and orthopedics by adapting mechanical and biomedical properties and pioneering implant designs with predictable features. This review primarily examines the current advancements in the additive manufacturing of tantalum and related alloys using the PBF-LB/M process. The analysis encompasses the evaluation of process parameters, mechanical properties, and potential biological applications. This will offer the reader valuable insights into the present state of PBF-LB/M for tantalum alloys.

## 1. Introduction

The process of additive manufacturing (AM) for metals involves the sequential fusion of raw materials, such as powder particles, wires, and sheets, by the use of various energy sources, ultimately resulting in the production of finished goods. Significant advancements have been made in this particular field of study, encompassing the enhancement of methodologies, equipment, and materials. Despite significant breakthroughs in the AM process, many technical obstacles still hinder the application and development of these AM process technologies [1,2,3,4]. Additive manufacturing of metals includes the layer-wise merging of raw material in the shape of powder particles, wires, and sheets, resulting in final products by applying several energy sources [5,6,7]. A wide variety of material options are available for AM, and the selection of material depends upon the manufacturing procedure and application where the manufactured part is used [8,9,10]. Due to tantalum’s high biocompatibility and its high-end safety applications, the research on Laser Powder Bed Fusion (PBF-LB/M) of tantalum is pressing. However, it faces significant challenges. This review aims to comprehensively examine the existing literature on the PBF-LB/M of tantalum alloys, presenting valuable insights for future research in this field.

### 1.1. Metal Additive Manufacturing

**Figure 1:**
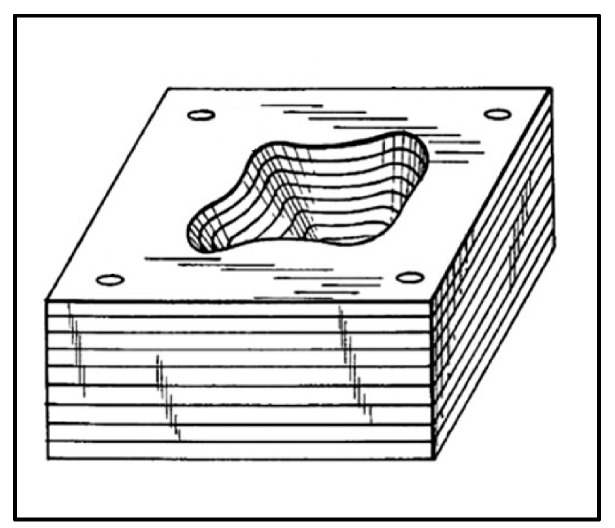
Manufacturing of die set by stacking the metal layers [11].

**Figure 2:**
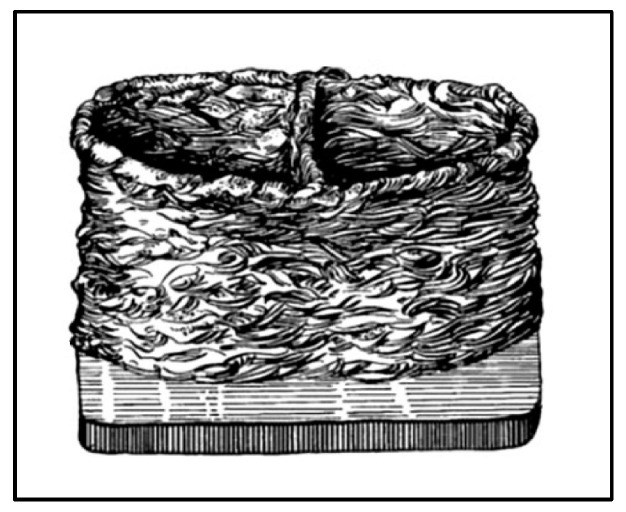
Welding layers are stacked and joined together to make the final object [14].

In earlier history, J.E. Blanther patented a process involving manufacturing a die set for pressing paper sheets using the ‘cut and stack’ approach [11]. Later, this approach was adopted by Dimatteo and Nakagawa for metal plates, and it was considered to be the very initial step toward metal additive manufacturing [12,13]. The first step includes cutting metal sheets using the milling cutter and then stacking metal sheets together to make an object. Then finally, the burr and rough edges are removed, as shown in Figure 1. Another earlier approach to metal additive manufacturing was centered on welding, consisting of layers of welding beads together to make a final object, as shown in Figure 2 [14].

**Figure 3:**
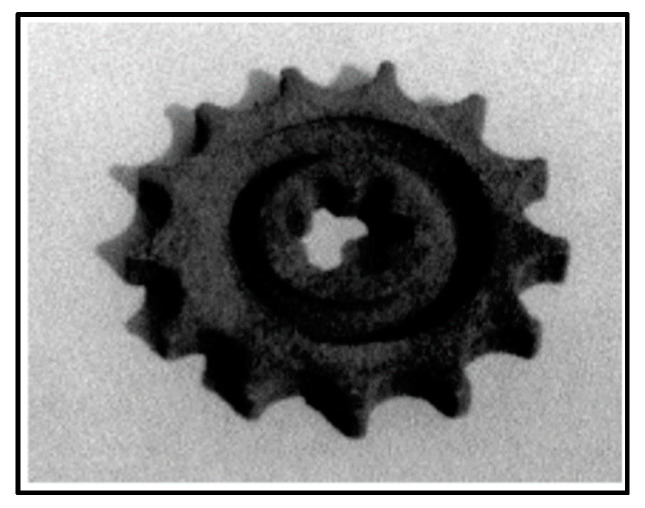
The first direct metal additive manufacturing part is approximately 7 cm in diameter [15].

Modern additive manufacturing started in the middle of 1990. Manriquez et al. manufactured a copper-solder mixture of metal parts using the laser sintering technique, as shown in Figure 3. The object was created by generating 72 layers, each representing one manufacturing step. Transient polymer binders were later commercialized for indirect laser sintering of metals [15]. The University of Texas started working on a direct laser sintering method [16,17,18]. Further, the latest systems, including second and third generations, were designed and established during the middle to the end of the 1990s [19,20,21]. Das et al. patented the direct SLS process; furthermore, it was researched and developed in Germany and Belgium [22]. The Institute for Laser Technology Germany adopted the Selective Laser Melting (SLM) term for the first time, and later it was patented in the USA. Kruths’s group at KU Leuven has also worked on metal additive manufacturing since 1991. MCP-HEK Germany split into companies, and SLM Solution was formed, dedicated explicitly to PBF-LB/M technology [23,24]. Electron Beam Powder Bed Fusion (PBF-EB/M) technology was successfully developed in 1997 with the collaboration of Chalmers University of Technology and Arcam AB. In 1997, Arcam AB was established in Sweden, and the initial batch of EBM12 was presented in 2002. ExOne started working on the process using a binder to fuse and join metallic powder into large dense parts; later, the model ProMetal RTS-300 was successfully installed and tested in 1998. To commercialize the powder bed processing technique for metallic elements, the company Layer-wise was acquired by the 3D system in 2014. Key innovations and improvements in metal additive manufacturing history and development are presented in Table 1.

### 1.2. PBF-LB/M of Bio-Materials

PBF-LB/M and Selective Laser Sintering (SLS) are techniques where powder bed fusion takes place through laser in which a specified amount of powder is spread on the bed by the movement of the sweeping head. Further scanning laser consolidates the successively deposited layers of metal powder. It is most suitable for pre-alloyed, pure, and multicomponent powders. PBF-LB/M is a rapidly developing powder bed fusion technique with a high-energy laser source to melt the whole metallic powder. High-melting pure metals and alloys may be printed with high density and enhanced mechanical properties, including Ti-Ta and Ni alloy powders [8,29,30].

The metallic materials that are frequently employed in biomedical applications include titanium alloys, cobalt-chromium alloys, stainless steel, tantalum alloys, gold, magnesium, gallium alloys, and iron. Each of these materials can undergo processing using one or more additive manufacturing methods. One of the primary applications of metals in the biomedical field is the utilization of orthopedic implants. The diverse range of material choices for these implants is a direct response to the evolving clinical demands. The predominant application of metallic materials in the biomedical field pertains to orthopedic implants. The diverse range of material choices stems from the need to accommodate evolving clinical demands. Certain applications necessitate inertness, such as in the case of Co-Cr alloys and gold. Conversely, other applications call for bonding with the host tissue, as seen with Ti alloys and Ta alloys. Lastly, there are instances where tissue growth and subsequent integration of the implant are desired, exemplified by Mg alloys and iron.

For a metal to be suitable for implantation, it is imperative that it possesses certain properties. These include biocompatibility, corrosion resistance, a high specific strength (which entails maximizing mechanical resistance while minimizing weight), high endurance strength (the maximum alternating stress that the material can withstand without experiencing fatigue failure over a given number of cycles), high impact toughness (the ability of the material to absorb energy through permanent deformation without fracturing), and low toxicity.

## 2. Effect of the Critical Process Parameters on the Properties of Ta Alloys by PBF-LB/M 

**Figure 4:**
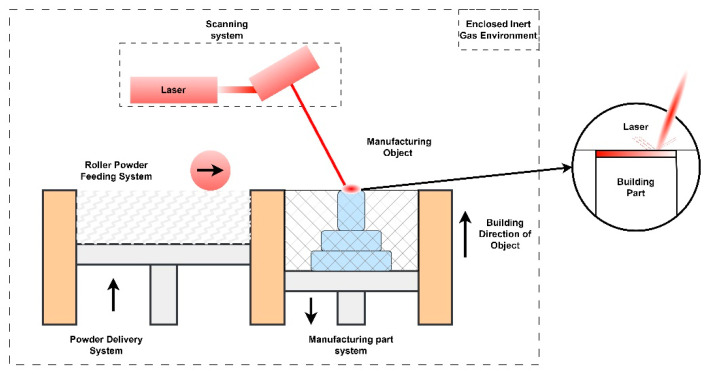
Illustration of PBF-LB/M principle [32].

The PBF-LB/M procedure involves the complete fusion of particles through the utilization of designated energy sources. After thermal energy impinges on the target, it fully melts the powder to an extendable depth according to the thermal energy applied. Subsequent scans of laser thermal energy consequences in the remelting of previously solidified particles and result in very efficient bonding of particles with a high-density ratio of metals and polymers. By using full melting techniques, entirely dense parts can be achieved with fewer porosity ratios. The full melting technique was successfully applied for many high-melting porous and non-porous metals [31]. A high-energy laser source is utilized in PBF-LB/M for the full melting of metal powders for Additive Manufacturing (AM), as shown in Figure 4. YAG laser or fiber laser is used. The input power, spot size, and power density can be varied according to metallic powder characteristics. Usually, it can be 100–400 W and 30–50 µm spot with power density over 5 × 10^6^ W/cm^2^.

The interaction area that changes dynamically between the high-energy beam and material is minimal during the material melting process. When the process starts, particles partially melt on the surface, and micro-melting of particles beneath the top surface due to gravitational interactions between the particles. Then, the metal powder particle absorption energy increased; thus, the surface melting quantity also increased correspondingly. After the formation of the molten metal pool, the melting metal powder reached a certain level. The powder is splattered as the laser beam advances due to the volume of the molten pool, the surface force, and the relative flow of the laser beam. The molten metal pool accelerates the heat transfer and helps bond the powder and its flow. In the laser trajectory, the metal particles situated ahead of the molten pool undergo a continual process of melting, while the liquid metal subsequently undergoes solidification. When the laser beam moves forward, a continuous solidification line is gradually formed along the beam path, and the forming is achieved.

The process control of PBF-LB/M is critical to manufacturing qualified products [33,34,35]. Powder quality, environment, scanning strategy, powder layer thickness, laser power, scanning speed, laser pulse frequency, scanning pattern, etc., influence heat distribution, and improper heat distribution may lead to some defects like holes and cracks, thus affecting the forming quality and performance.

**Figure 5:**
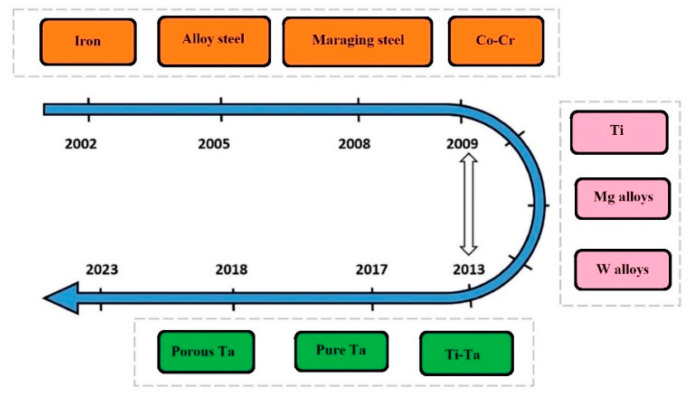
Development of PBF-LB/M according to metals [2].

Fraunhofer Research (Germany) put forward the PBF-LB/M technology in early 1995. MCP HEK company launched the first PBF-LB/M system in 2003. PBF-LB/M is considered one of the most suitable choices for metals ranging from aluminum, nickel, and titanium to tantalum and tungsten alloys. Figure 5 shows the evolution of PBF-LB/M according to metals, which began with iron-based powder and then further researched into the alloy steel, Mg alloys, and porous and non-porous Ta alloys [24,36,37,38,39,40,41,42]. The group led by Prof. Jean-Pierre Kruth started working on biomedical materials, including Ta alloys, for dental implants and bone replacements using PBF-LB/M in 2013.

Tantalum, named by Anders Gustaf Ekeberg, who discovered the metal in 1802, originates from the word “tantalizingly” and “tantalus”, which mean that it is difficult to be separated from the niobium and father of Niobe, respectively [43]. It has broad applications in the aerospace, medical, electronics, and nuclear industries [44,45]. The physical properties of pure Ta are shown in Table 2. Ta has excellent corrosion resistance, a high melting point, high thermal resistance, and extraordinary biocompatibility, based on which it may be ranked Ta > Ti > Cr on a functional superiority scale [46,47].

**Figure 6:**
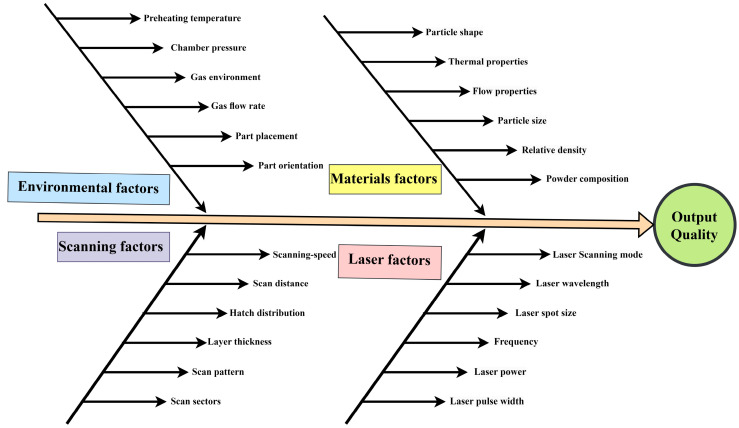
Influence of the critical process parameters on PBF-LB/M output quality [55].

PBF-LB/M of tantalum alloys has been a hot topic of research owing to its high biocompatibility and low cytotoxic and application in high-end safety applications. The medical implant of tantalum alloy by PBF-LB/M process is promising, especially in artificial joint replacement surgery, dental, and orthodontics [48,49,50]. On the other hand, although significant research has been conducted on the PBF-LB/M of Ti alloys, much less has been performed on β-Ti alloys with higher mechanical properties, higher corrosion resistance, and lower modulus of elasticity than other Ti alloys. Tantalum, as a β stabilizer, is considered the best choice for bio applications. In addition, additive manufacturing of porous and bio-metallic materials directs the medical industry’s attention and enables the fabrication of intricate components that traditional methods cannot do. Porous Ta is desired for biomedical applications due to its excellent osseointegration properties and high biocompatibility [51,52,53,54]. The influence of the critical process parameters in PBF-LB/M is illustrated in Figure 6 [55,56,57,58]. Optimization of several parameters, including laser (power, spot size, wavelength), metallic powder (size, type, physical properties), scanning parameters (scanning speed, distance, hatch spacing), and environmental parameters (pressure, pre-heating temperature, gas flow) is needed to ensure surface quality.

As one of the most critical factors, energy density $E_{d}$ influences the physical densification, which changes the quality of the part fabricated via PBF-LB/M. It is defined as in Equation (1)

(1) $$E_{d}=\frac{P}{vhl}$$

where *P* is the power of the laser, v represents scanning speed, h represents hatch spacing, and *l* is layer thickness. Scanning speed represents the fabrication time of parts and is defined as the rate at which the scanning path traverses the material. Higher scanning speed reduces production time, but scanning speed can be optimized with laser intensity and other parameters, i.e., the higher scanning speed requires additional optimization. Low laser energy density and high scanning speed may partially melt the powders. In contrast, low scanning speed and high laser energy density may result in sputtering and over-melting powder. Both result in poor quality. Hatch spacing influences the bonding of melting layers of powders, affecting the surface roughness and porosity of the output product. In the context of the selective laser melting process, it is crucial to achieve a harmonious equilibrium between the power and scan speed parameters. When employing a low scanning speed, the significance of heat gradients becomes apparent since localized solidification may result in the formation of fissures. Conversely, the utilization of high speed necessitates a substantial power input, leading to the emergence of heat transfer-related phenomena such as delamination or balling.

**Figure 7:**
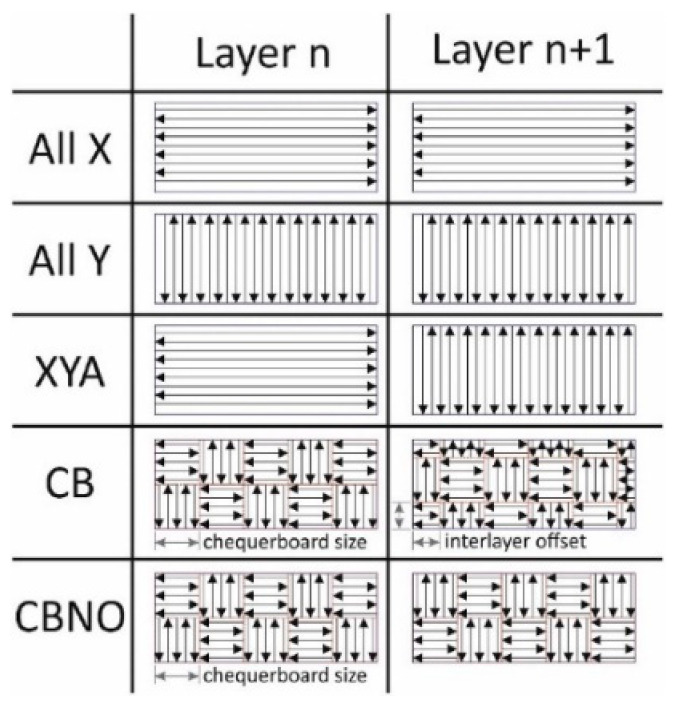
Schematic representation of laser scanning strategies of the PBF-LB/M process [61].

Bonding and overlapping of neighboring melted tracks are highly influenced by hatch spacing in PBF-LB/M fabricated parts, consequently affecting surface roughness and porosities [59,60]. The scanning path has several patterns, including straight, strips chessboard, meanders, chequerboard, zigzag, etc., as shown in Figure 7. The scanning path influences the PBF-LB/M parts quality by affecting and taking care of the thermal properties, powder properties, and laser parameters [61,62].

**Figure 8:**
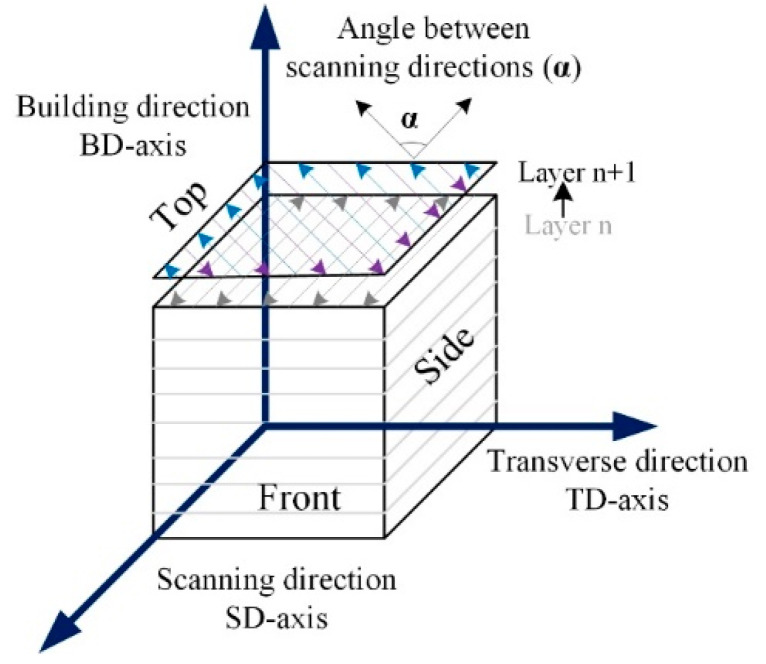
Illustration of the coordinate system with SD, TD, and BD indicated, and the different cross-section views for microscopy. The arrows indicate the movement of the laser, and α is the rotation angle of the scanning direction between two consecutive layers *n* and *n* + 1 [63].

Thijs et al. [63] conducted an experimental study on PBF-LB/M of pure Ta using particle size 13–26 µm. The microstructures, mechanical properties, and thermal profiles around the melt pool were modeled using a pragmatic model. The influence of the scanning strategy was evaluated on pure Ta parts, and optimization for high-end dense parts was accompanied. The density was calculated using the Archimedes principle by relating the theoretical density of 16.6 g/cm^3^. Three different scanning schemes were adopted using long bidirectional vectors, while in between the adjacent layers *α*, the scanning direction angle is chosen to be 0°, 60°, and 90° respectively, as shown in Figure 8. The presence of both morphological and crystallographic texture in PBF-LB/M of Ta specimens contributes to the anisotropy of yield strength.

**Figure 9:**
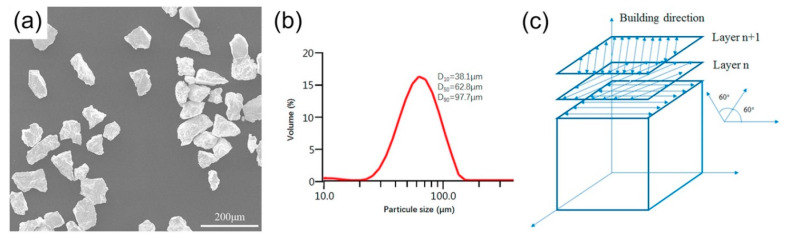
(**a**) Morphology of pure Ta powder particles; (**b**) particle size distribution of pure Ta; (**c**) schematic diagram of scanning mode of pure Ta [64].

Zhou et al. [64] studied the effect of parameters on densification, mechanical properties, and microstructure in PBF-LB/M of pure Ta. Experiments were performed using a 500 W Yag fiber laser that contains 80 µm spot size in an argon environment with no less than 120 ppm oxygen contents. Micropores and discontinuous paths were observed, which may be connected to the Magroni convection and balling effect. Interlayer thermal microcracks were observed at lower scanning speeds owing to the balling effect and thermal stress. Surface morphologies and scanning paths are shown in Figure 9.

The impact of process parameters on the properties of (Ta) and its alloys is of significant importance. An extensive literature review has been conducted to gather relevant information, resulting in the development of Table 3. This checklist serves as a valuable resource for readers seeking helpful data on Ta alloys produced through the PBF-LB/M process.

### 2.1. Porosity and Density

**Figure 10:**
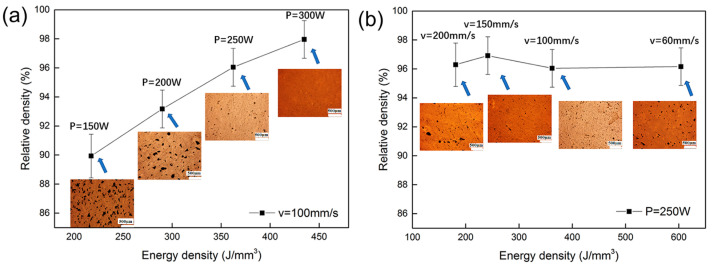
Energy density effects on densification of components of pure Ta fabricated by PBF-LB/M with constant (**a**) scanning speed and (**b**) laser power [64].

The porosity and density of a material are significant factors that have a substantial impact on the mechanical strength of the manufactured components, hence playing a critical role in their application throughout service. Figure 10 shows the relative density of the pure Ta part fabricated by PBF-LB/M with scanning speed, laser power, and optical micrographs. A positive correlation between energy density and the relative density of pure Ta can be observed when the scanning speed is fixed at 100 mm/s. Many pores were found when the energy density was 217.39 J/mm^3^ (*P =* 150 W), and 10.07% porosity was observed in the output PBF-LB/M part. This phenomenon is associated with insufficient energy, which may cause an increment in the unbalanced viscosity of the liquid pool [66,76]. A higher energy density of 362.32 J/mm^3^ (*P* = 250 W) results in a pore-less structure with highly dense parts, approximately 96% dense, as shown in Figure 10a.

However, the correlation between input energy density and densification is not proportional under all conditions, as shown in Figure 10b, at 181.15 J/mm^3^ (*v* = 200 mm/s), irregularly formed micro-pores were observed in the interlayers. The findings of this study indicate that micro-pores were observed to form at greater scanning speeds. As a result, it can be inferred that lower energy levels are related to inter-ball pores due to the presence of discontinuous tracks, Marangoni convection, and the balling effect.

Livescu et al. [65] fabricated pure Ta cubes by performing experiments on the EOSINT M280 DMLS system by Gmbh (EOS) furnished with a fiber laser of 400 W. As shown in Table 4, when the energy density increases from 133.48 to 209.28 J/mm^3^, the percentage of area porosity dramatically drops to 0.46 from 2.85%. A decrease in hatch spacing results in a reduction of porosity. Qualitatively, porosity exhibits an inverse relationship with increasing overlay, owing to the high chance of boundary zones re-melting between the bands where it is much expected that solidification defects occur commonly. The research concludes that the EOSINT M 280 DMLS system successfully produced totally dense tantalum with occasional porosity (0.018% area fraction) after optimizing the machine build settings.

To visualize the effects of process parameters on the quality of the Ti-50Ta sample’s surface, the samples’ densities were measured using the analytical balance on the base of the Archimedes principle. Absolute density was calculated using Equation (2). Two groups of samples were utilized, one of which was weighed in deionized water. In contrast, the other one was weighed in dry condition.

(2) $$\rho_{abs}=\frac{m_{air}}{m_{air}-m_{water}}\times \rho_{water}$$

where $\rho_{abs}$ absolute density of the sample, $m_{air}$ sample mass while weighing in dry condition, $m_{water}$ sample mass while weighing in deionized water, $\rho_{water}$ deionized water density. Relative density $\rho_{relative}$ can be calculated using Equation (3).

(3) $$\rho_{relative}=\frac{\rho_{abs}}{\rho_{theoretical}}\times 100\%$$

**Figure 11:**
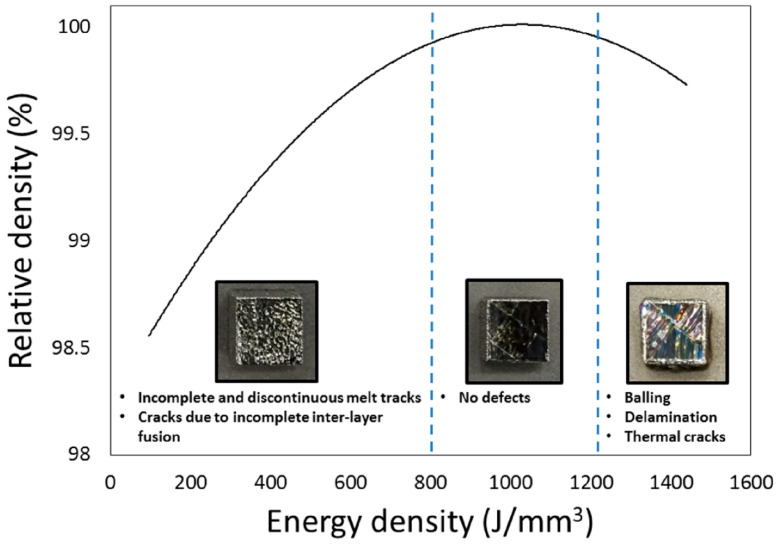
Correlation concerning energy density and relative density of Ti-50Ta samples by PBF-LB/M [77].

The fabricated samples were studied using an optical microscope to visualize the microstructure under varying conditions and parameters. The relationship between the relative density and macrostructure of Ti-50Ta samples by PBF-LB/M is shown in Figure 11. The energy density of less than 800 J/mm^3^ generates the melting pole but not enough to fully fuse the in-depth layer of the powder, which primes cracks and porosity. Excessive energy also results in the parts’ delamination, balling, and cracking. The optimization may be obtained according to process factors, metallic material, and environmental parameters [77].

**Figure 12:**
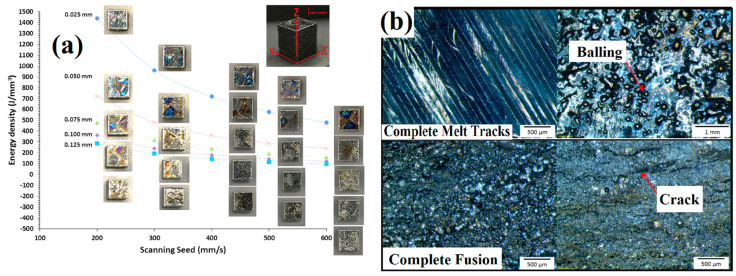
(**a**) Energy density effect on surface morphology along XY-plane of Ti-50Ta by PBF-LB/M, (**b**) comparison of full melting and fusion tracks with the surface defects [67,77].

Samples were further prepared for optical micrographs in XY planes to visualize the energy density effect on the surface morphologies. As shown in Figure 12a, samples of XY planes are displayed according to the energy density and scanning speed; Figure 12b depicts the comparison of complete melt tracks and complete fusion with the balling effect and different cracks [77].

The generation of thermal gradients across the layers results in delamination, which can be connected to the incomplete fusion of particles because of low penetration energy. Furthermore, the melting process generates cracks due to shear stress. Stresses were developed due to the melting and cooling process, and it has been reported that tensile stress was observed at the bottom and top of the fabricated samples while compressive in between segments [36,78].

**Figure 13:**
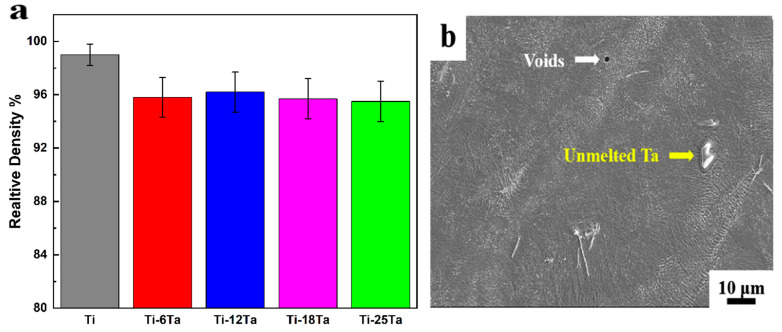
(**a**) Relative densities of Ti-Ta alloys by PBF-LB/M and (**b**) SEM image of Ti-25Ta alloy fabricated by PBF-LB/M [79].

**Figure 14:**
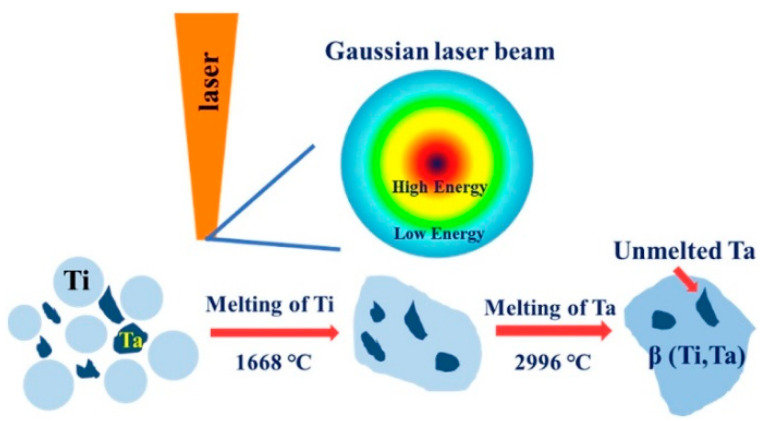
Illustration of the formation of un-melted Ta particles in PBF-LB/M of Ti-Ta alloys [79].

PBF-LB/M of titanium-tantalum (Ti-Ta) alloys have a significant impact on particle melting behavior, as demonstrated in Zhao’s [79] research findings. Experiments were conducted on Ti-6Ta, Ti-12Ta, Ti-18Ta, and Ti-25Ta to visualize the melting behavior of the melting pole. Figure 13a shows their relative densities. There exist un-melted Ta particles that are shiner than Ti because of their higher atomic number, as shown in Figure 13b. This is because Gaussian energy distribution results in temperature not reaching the melting temperature of 2996 °C at some places, as illustrated in Figure 14. There is no observance of macrocracks near the un-melted Ta particles, which depicts the homogeneous bonding between Ti and Ta. The pre-alloyed powder also resulted in a homogenous microstructure and improved fatigue performance in PBF-LB/M and PBL-EB/M [80,81].

**Figure 15:**
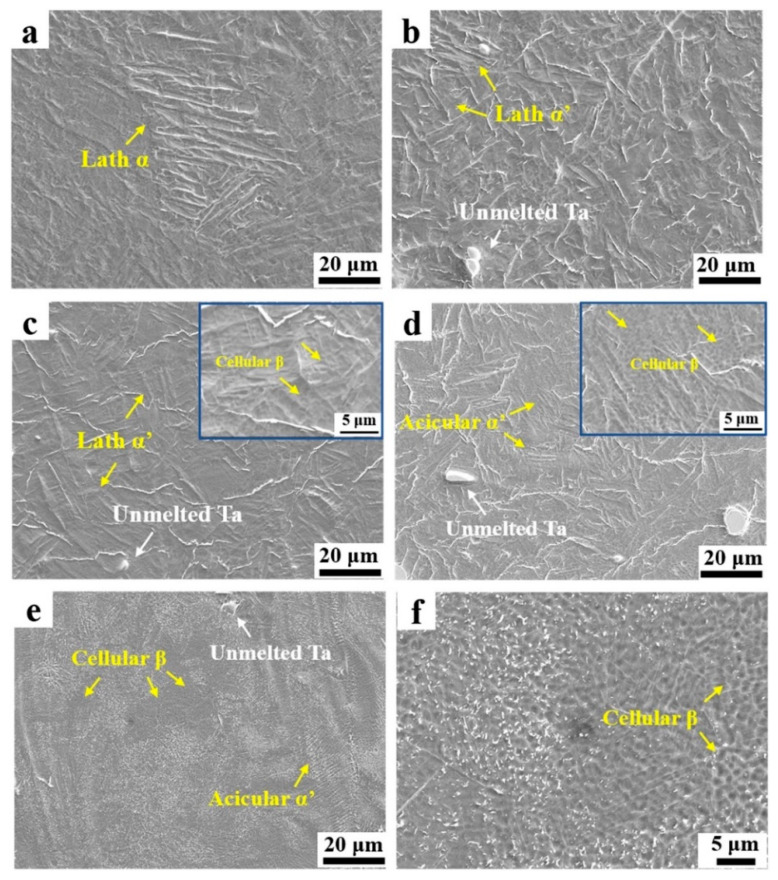
Microstructure of Ti-Ta alloys by PBF-LB/M with various Ta contents (**a**) Ti, (**b**) Ti–6Ta, (**c**) Ti–12Ta, (**d**) Ti–18Ta, (**e**) and (**f**) Ti–25Ta, cellular β phase appears at 5 µm as shown inset of (**c**,**d**) [79].

Figure 15 illustrates the effect of the Ta ratio on the microstructure of PBF-LB/M-produced Ti-Ta alloys. Figure 15a–c depicts a typical lath particle α with a length of ~60 µm in Ti, while martensite α′ with a lath structure is present in both Ti-6Ta (b) and Ti-12Ta (c) alloys. In the Ti-12Ta (c) alloy, colonies of parallel and finer lath structures formed. Due to its low concentration, the β phase cannot be found in the Ti-6Ta (b) alloy, whereas the cellular β phase is present in the Ti-12Ta alloy in-set of (c) at 5µm. The Ti-18Ta (e) alloy contains acicular martensitic α′ and cellular β grains with a width of ~1 µm (d), whereas the Ti-25Ta (f) alloy contains more cellular β grains and fewer α′ grains. Due to the low diffusion coefficient of Ta in the Ti matrix, the planar structure becomes unstable, and the cellular structure is induced. Undercooling during solidification in PBF-LB/M-processed Ti-Ta alloys causes the destabilization of the planar solidification due to impurity redistribution, thereby facilitating the transition from a planar to a cellular solidification mode.

Ta contents impacted the solidification’s starting temperature, and owing to this effect, multiple microstructural behaviors were observed. As the Ta contents increase, the percentage of β grains also increases. Zhao et al. [79] concluded that microstructure evolution could be Lath α grain → lath α′ grain → acicular α′ grain → acicular α’ + cellular β grains.

Several variables contribute to the lower energy levels observed in inter-ball pores. These aspects include the presence of discontinuous tracks, the occurrence of Marangoni convection, and the process of balling. The emergence of delamination, balling, and cracking is a direct result of an excessive input of energy. The occurrence of layer delamination can be attributed to the existence of temperature disparities. The selection of optimal settings for PBF-LB/M of Ta and its alloys is contingent upon the specific composition and equipment employed.

### 2.2. Microhardness

The assessment of microhardness is of utmost importance in the evaluation of the Laser Powder Bed Fusion (PBF-LB/M) process for tantalum (Ta) alloys. Microhardness is a vital characteristic, particularly in applications where excellent wear resistance is necessary. In general, highly dense parts without the formation of microspores and cracks result in high values of microhardness [82,83]. Vickers microhardness was measured on the polished samples with a load of 0.98 N and a dwell time of 10 s. Multiple points were selected for each piece. Ta parts produced from PBF-LB/M without any post-processing result in higher microhardness values than conventionally manufactured parts, as shown in Table 5.

**Figure 16:**
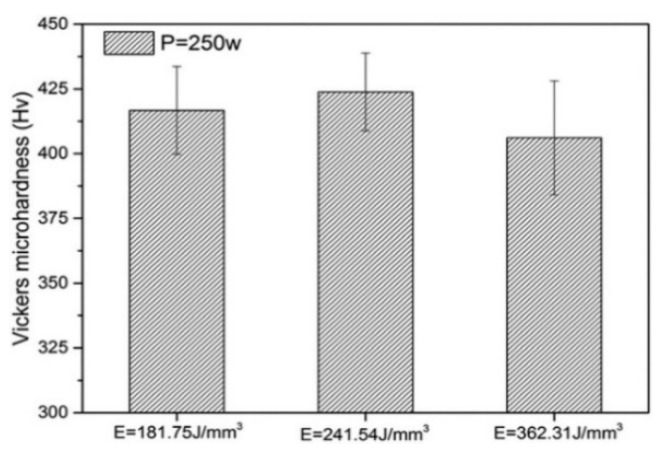
Vickers microhardness of pure Ta parts by PBF-LB/M [64].

The microhardnesses of pure Ta samples of PBF-LB/M using a 250 W laser (FS271M Farsoon, Inc., Changsha, China) with three different energy densities are shown in Figure 16. The highest microhardness was found at medium energy density (241.54 J/mm^3^). Increasing or decreasing the energy density above or below this value decreased the microhardness. This phenomenon can be explained by two factors: the considerable collapse when the stress acted on the sample’s surface due to the lower densification at high scanning speed and the grain coarsening due to thermal accumulation and overheating at low scanning speed [64].

**Figure 17:**
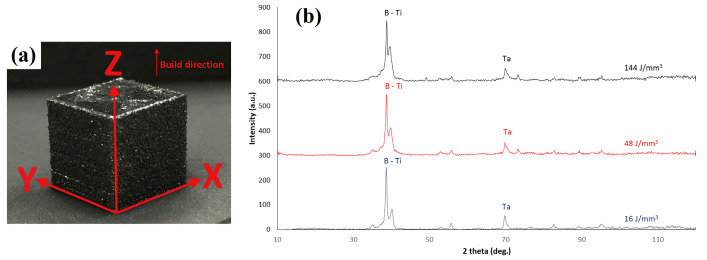
(**a**) Build orientation of the samples of Ti-50Ta by PBF-LB/M (**b**) XRD patterns at various energy densities [67].

**Figure 18:**
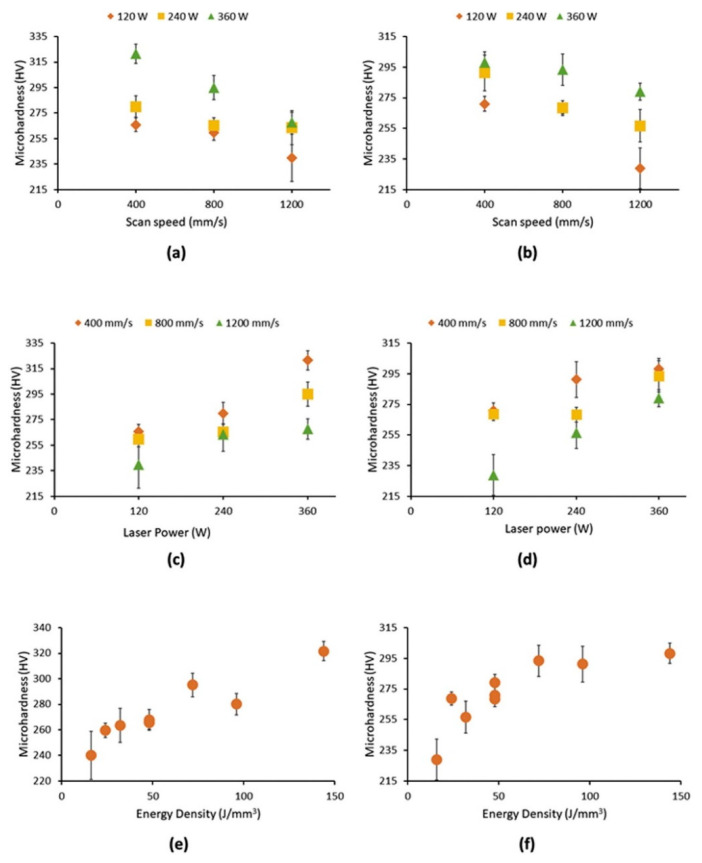
Microhardness deviation of PBF-LB/M Ti-50Ta specimens in XY and YZ planes under varying parameters, (**a**) scanning speed XY, (**b**) scanning speed YZ, (**c**) laser intensity XY, (**d**) laser intensity YZ, (**e**) energy density XY (**f**) energy density YZ [67].

Sing et al. [67] studied the effects of process parameters on the microhardness of Ti-50Ta parts manufactured by PBF-LB/M ; the build orientation of the fabricated samples of Ti-50Ta is shown in Figure 17a. Microhardness values along XY and YZ directions are expressed in Figure 18. High laser intensity and slow scanning speed increase relative density and decrease the porosity of fabricated samples, resulting in higher microhardness and strength. XRD analysis in Figure 17b shows β-titanium in all the samples. Hence, the variance in microhardness may be caused by the samples’ varying porosities and tantalum concentration.

Ta contents are significant in defining the mechanical properties of Ta-Ti specimens, as shown in Table 6. With the increased percentage of Ta, Mechanical properties, including yield strength, ultimate tensile strength, and microhardness, are linked to grain refinement and solid solution strengthening.

**Figure 19:**
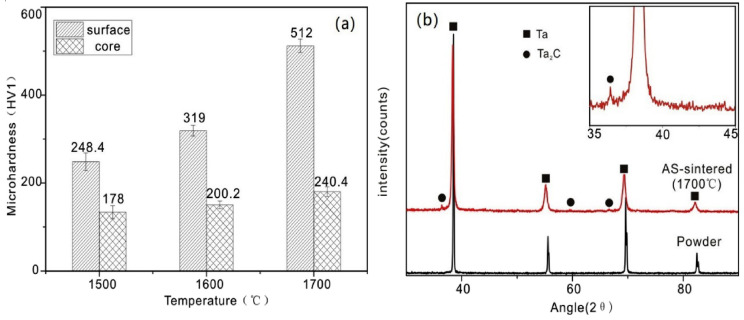
(**a**) Microhardness at the surface and core of pure Ta parts fabricated at various spark plasma sintering temperatures, (**b**) comparison of XRD maps of Ta powder and AS-sintered Ta [87].

Similar results can be found in the research on pure Ta processed by spark plasma sintering technique [87]. Microhardness tests on polished samples of Ta using a load of 9.8 N were conducted by employing an HVS-1000 hardness (Laryee Technology, Beijing, China) indentation machine. Microhardness values at the surface and core show an increasing trend concerning sintering temperature, as shown in Figure 19a. At the sintering temperature of 1500 °C, the hardness at the surface is 248.4 HV_1_, about 38% of that at the core 178 HV1. At 1700 °C, the hardness at the surface and core are 512 HV_1_ and 240.4 HV_1,_ respectively, which shows a difference of more than double. Higher surface hardness corresponds to the decarburization layer generated on the surface of Ta during the sintering process, which can be seen from XRD analysis, as shown in Figure 19b. Higher sintering temperature results in higher density values, lower porosity, and higher microhardness.

### 2.3. Tensile Strength

**Figure 20:**
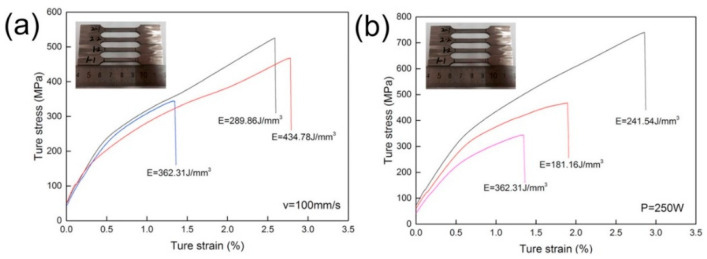
Stress-strain curves of pure Ta parts by PBF-LB/M under different energy densities (**a**) at a speed of 100 mm/s (**b**) at a laser intensity of 250W [64].

Energy density and optimization of process parameters resulted in the formation of highly dense parts. However, it was noted that there was a significant fluctuation in the ultimate tensile strength (UTS) of the produced parts. Zhou et al. [64] conducted experiments on Ta samples to visualize the process parameters’ effect on microstructural and mechanical properties. As shown in Figure 20a, when the scanning speed is 100 mm/s, an energy density of 289.86 J/mm^3^ results in a tensile strength of 525.47 MPa; a further increase in energy density results in lower tensile strength owing to the extreme melting of powders, which in turn to the balling phenomena. It is similar in the case of adopting a laser power of 250 W. The interlayer porosity shown in the inset of Figure 12b resulted in a lower ultimate tensile strength of 575.65 MPa at the energy density of 181.15 J/mm^3^ than that of 739.36 MPa at the energy density of 241.54 J/mm^3^. The changes in laser power and scanning speed had differing effects on the ultimate tensile strength, although the energy densities were close, i.e., 241.54 J/mm^3^ and 289.86 J/mm^3,^ as shown in Figure 20a,b. It can be figured out that increasing the scanning speed and laser power simultaneously results in higher tensile strength of Ta parts. High tensile strength is attributed to the grain enhancement, which is due to the high solidification rate owing to the enhancement of the scanning speed of the laser source. It was observed that Ta parts fabricated from the PBF-LB/M process owned a higher tensile strength of 739 MPa compared to those by casting (205 Mpa) [84], powder metallurgy (310 MPa) [84], soft annealing (200–390 MPa) [88] and cold working (200–1400 MPa) [88].

**Figure 21:**
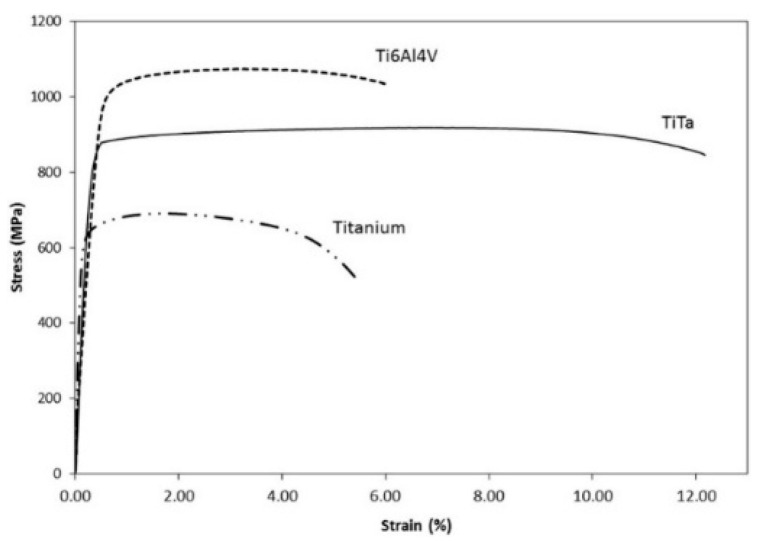
Stress-strain curves of Ti-Ta parts by PBF-LB/M and comparison with pure Titanium and Ti6AL4V [66].

Using Instron Static Tester Series 5569 machine by Instron, Norwood, NA, USA, tensile tests were performed to obtain the engineering stress-strain curves shown in Figure 21 for comparing the tensile strength of pure Ti, Ti6Al4V, and Ti-50Ta samples by PBF-LB/M along the build direction XY-plane by applying a load of 50 kN and keeping strain rate at 1 mm/min. Young’s modulus of the Ti-50Ta was found to be the lowest (75.77 GPa) compared to CPTi (111.59 GPa) and Ti6Al4V (131.51) [66]. The elastic modulus of Ti-50Ta may be calculated by the volume fractions and modulus of the constitution phases irrespective of the grain size [89], and it was reported by Edward et al. that the lowest young modulus is the β phase while the highest value is the ω phase [90]. Ti-50Ta samples show higher ductility, as depicted by higher elongation yield compared to pure Ti and Ti6Al4V, as shown in Figure 21. This is due to the absence of α′ martensitic phase, a principal constituent of strain hardening in Ti6Al4V.

**Figure 22:**
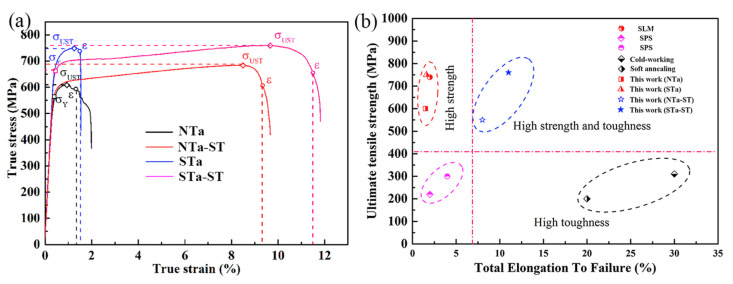
(**a**) Stress-strain curves of STa (spherical), NTa (Non-spherical), STa-ST (spherical annealed), NTa-ST(non-spherical annealed) samples by PBF-LB/M (**b**) UTS and total elongation up to failure for pure Ta samples fabricated via various process [91].

A study by Zhao et al. [91] was conducted on pure Ta samples of spherical powder (Sta), non-spherical powder (NTa), and annealed samples of spherical and non-spherical (STa-ST, NTa-ST), and it was observed that improved ductility with high strength can be achieved by selecting suitable powder in PBF-LB/M. As shown in Figure 22a, Non-spherical Ta and spherical Ta samples showed an ultimate tensile strength of 600 and 750 MPa, respectively, but lacked ductility. Increasing grain size and reducing the relative density of the STa sample in comparison to the NTa sample results in low tensile strength. The ultimate tensile strength values of all the PBF-LB/M-processed pure Ta samples, regardless of the kind of raw powders, are higher than the corresponding values of the conventionally manufactured samples, as shown in Figure 22b.

**Figure 23:**
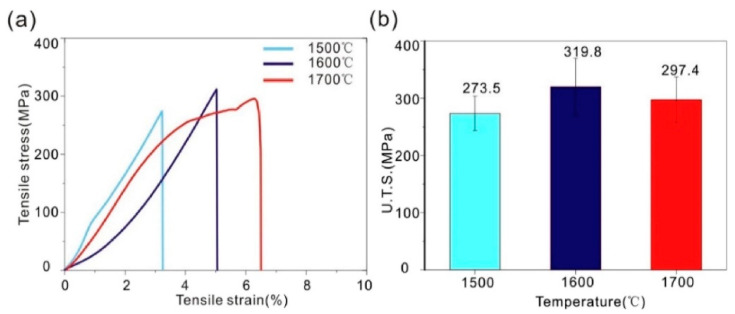
(**a**) Stress-strain curves and (**b**) UTS of pure Ta fabricated by spark plasma sintering at 1500 °C, 1600 °C, and 1700 °C [87].

In the study by Dong et al. [87], tensile tests were conducted on pure Ta fabricated by spark plasma sintering. Tensile tests of sintered samples at various sintering temperatures are displayed in Figure 23 to evaluate the mechanical properties of pure Ta at room temperature. As shown in Figure 23a, when the sintering temperatures are 1500 and 1600 °C, elongations are 2.3% and 5.1%, respectively, without necking observed, which means brittle fracture. While at 1700 °C, it shows a low ductility with necking observed and an elongation of 6.4%. As shown in Figure 23b, UTS are 273.5, 319.8, and 297.4 MPa, respectively, when the sintering temperatures are from 1500 to 1700 °C.

From the perspective of powder metallurgy, Ta powder particles are enormously affected by sintering temperature, which further influences mechanical and physical properties. In the case of low temperature during the sintering process, the powder is partially melted and solidified with high-density ratios due to insufficient bonding energy, which further results in more pores and, ultimately, more inferior mechanical properties. On the other hand, if the sintering temperature is high, bonding will be increased, and fewer pores will be generated, resulting in top-dense parts and, eventually, upshots with high mechanical strength and toughness properties. However, very high sintering temperature leads to over-melting and grain oversizing, resulting in splashing and balling phenomena and reduced strength of the manufactured parts.

## 3. Porous Ta as a Biomedical Material for PBF-LB/M

**Figure 24:**
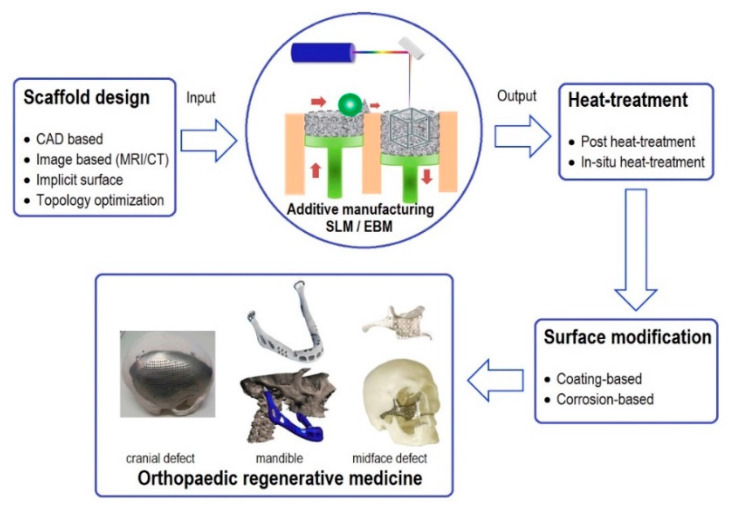
Illustration of the process of orthopedic regenerative body parts of porous metals from CAD to AM, heat treatment, and surface treatment [98,99,100].

In orthopedics, the design of implants and bone scaffolds should imitate the biomechanical properties of the host bone. This issue can be tackled by developing porous metals ideal for bone repair and replacement since their porosity and stiffness can be controlled. Porous metals depict excellent Osseo-integration properties with good in-growth of bone tissues [92,93,94,95]. Controlling porous metals is challenging, and the development of additive manufacturing makes it possible for the latest innovation and the fabrication of porous metals for biomedical applications. Researchers have continuously worked on topology optimization techniques to achieve porous metals’ internal architecture and mechanical properties according to the need. Figure 24 illustrates the process of making orthopedic regenerative parts of porous metals from the CAD to final products via AM, heat treatment, and surface treatment [96,97].

**Figure 25:**
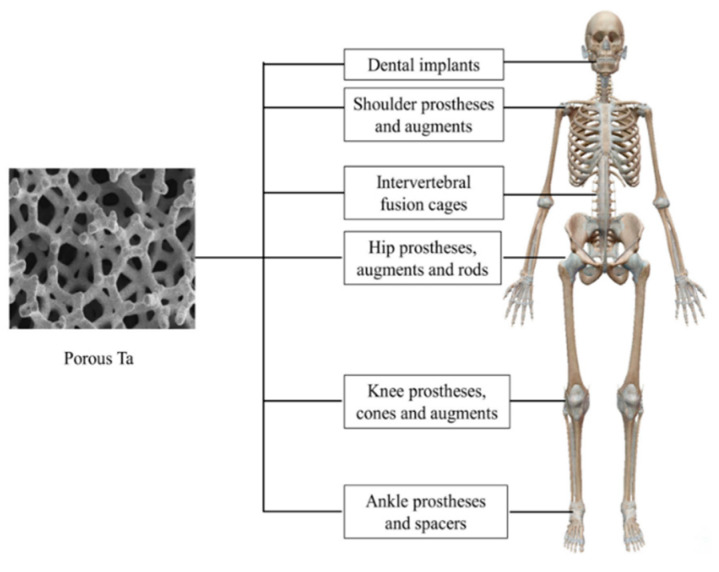
Application of Porous Ta in the human body [102].

Researchers have successfully manufactured and implanted the porous Ta and achieved in-animal growth. Porous Ta scaffolds and implants can now be fabricated by Electron Beam Powder Bed Fusion (PBF-EB/M), laser engineering net shaping (LENS), Laser Powder Bed Fusion (PBF-LB/M), etc. Compared to other manufacturing processes such as CVD, more excellent performance can be achieved with acceptable cost-efficiency, reduced time, and material consumption. The macrostructure and microstructure of porous Ta can be accurately controlled using AM technology by the manufacturing procedure and design specifications. The porous Ta scaffolds produced via AM exhibit acceptable fatigue strength and load-bearing capability [101]. Additionally, various modification techniques have been adopted to improve porous Ta’s bioactivity and antibacterial properties in preparation for its potential use in bone tissue creation. Orthopedics and dentistry have used porous Ta-based implants or prostheses widely. The applications of porous Ta in the human body are shown in Figure 25.

Some researchers found failure for multiple reasons, including the implanted parts’ brittle fracture [103,104]. Porous Ta-based implants or prostheses offer primary stability due to the high friction coefficient. It is worth noting that higher pore size and porosity are associated with acceptable biological performance, but there is an inverse relationship with mechanical strength. Therefore, getting an appropriate balance between biological and mechanical properties is still challenging. PBF-LB/M of Porous Ta for biomedical applications still needs further research regarding the parameter’s optimization concerning the application.

## 4. Summary

1. It may be ranked Ta > Ti > Cr on a functional superiority scale for the application of medical implants mainly due to the extraordinary biocompatibility of Ta. Ta with a combination of Ti warrants excellent mechanical properties, and the contents of Ta are critical. PBF-LB/M’s medical implant of Ta alloy is promising and primarily used for artificial joint replacement surgery, dental, orthodontics, etc. Many factors, including laser power, metallic powder size, scanning speed, pre-heating temperature, gas flow rate, part orientation, etc., significantly affect the quality of Ta alloy parts by PBF-LB/M. Increasing scanning speed may reduce production time, but it needs to be optimized with laser intensity and other parameters.
2. The correlation between energy density and densification is not proportional under all conditions; there is an optimization for favorable results. Ta alloy parts by PBF-LB/M show higher microhardness than those fabricated using conventional processes. Higher energy intensity and slow scanning speed result in higher microhardness, which can be connected to the lower porosity and higher relative density.
3. Ta alloy parts fabricated by PBF-LB/M have higher tensile strength than those manufactured using conventional processes. Increasing laser power and scanning speed simultaneously leads to the higher tensile strength of pure Ta due to finer grain structure.

Compared to Ti and Ni, more research needs to be conducted for the PBF-LB/M of Ta. Porous Ta depicts excellent Osseo-integration properties with good in-growth of bone tissues, and the fabrication of porous Ta by PBF-LB/M is promising but challenging. The lattice structure of Tantalum manufactured by PBF-LB/M exhibits notable attributes such as exceptional strength and biocompatibility, rendering it suitable for manufacturing purposes. Biomaterials have the capability to undergo additive manufacturing processes through PBF-LB/M on the surface of non-biomedical materials, particularly Tantalum alloys. This manufacturing approach has demonstrated favorable biocompatibility when compared to Titanium and other alloys. The current study on s PBF-LB/M of tantalum mostly concentrates on process optimization to achieve high-quality output. However, there is a dearth of studies investigating the melt pool and thermal modeling aspects of PBF-LB/M for porous Ta. The flow of argon gas is a crucial factor in the PBF-LB/M process for all materials. There is a research gap in the computational fluid dynamics (CFD) modeling of argon flow during PBF-LB/M processes that need to be addressed.

## Figures and Tables

**Table 1:** Metal additive manufacturing history and development.

Research Work	Year	Detailed Description
Cut and stack	1892	Blanther et al. The contour of relief maps was manufactured US Patent No. 473901 [11]
Weld overlay	1925	Baker et al. Methodology to manufacture decorative products US Patent No. 1533300 [14]
Stacking of metal sheets to make an object	1976	DiMatteo et al. Generation and construction of 3D objects US Patent No. 3932923 [12]
AM technology product development	1987	Expansion of lightweight materials for AM processing Chuck Hyull (Co-Founder of 3D systems) [25]
72 layers object formed by laser sintering copper-solder	1990	Manriquez et al. Utilization of Selective Laser Sintering (SLS) technique for Binary Metallic Powder [15]
Metal laser melting (direct method)	1991, 2014	Developed at KU Leuven by Kruth’s group [24]
EBM powder bed	1997	EBM technology was developed with the collaboration of Arcam AB and Chalmers University of Technology, Gothenburg [26]
Directed energy deposition approach	First machine shipment, 1998	LENS was invented by Optomec (1990s)
ExOne was founded as Extrude Hone	ProMetal RTS-300, 1998	Worked on the process using a binder to fuse and join metallic powder into large dense parts
Ultrasonic Additive Manufacturing	1991	Ultrasonic consolidation of aluminum tooling [27]
Institute for Laser Technology in Germany	2003	Gasser, Meiners, Wissenbach, laser beam scanning the surface of complex geometry part US Patent 6534740 [23]
Direct SLS patented	2004	S. Das, J.J. Beaman, Direct Selective Laser Sintering of Metals, US Patent [28]
NORSK TITANIUM	2007	Pioneer in AM machine technology by using the traditional plasma arc welding process
Irepa Laser EasyCLAD	2010	LMD through a powder-fed mechanism via the nozzle similar to LENS (Wohlers Reports 2016)
SCIAKY, Wire Electron Beam Deposition	2014	SCIAKY introduced its Wire Electron Beam Deposition system as a solution for producing Titanium blanks
DESKTOP Metal and HP	2016	DESKTOP METAL and HP introduced metal BJT technology, promising increased productivity compared to PBF
Optomec Metal Printing on Plastic	2017	Optomec develops a system for printing metal onto 3D-printed plastic parts, enabling hybrid additive manufacturing
Renishaw, Materialise	2020	In-situ sintering for powder bed fusion AM, Automated support removal for metal AM, Automated support removal for metal AM
Fabrisonics, SonicLayers 600	2023	Hybrid platform additive and subtractive, UAM and CNC milling platform
Speed3d partnership with the University of California, Irvine	2023	Cold spray method for additive manufacturing of metals through metal powder particles blasted at high speed in a suspended environment.

**Table 2:** Physical properties of Ta.

Melting point	2996 °C
Boiling point	5425 °C
Specific heat at 0 °C	0.033 Cal/g/°C
Recrystallization temperature range	1000–1375 °C
Heat of fusion	34.6–41.5 Cal/g
Ultimate tensile strength	276 (MPa)
Percentage elongation	50

**Table 3:** Latest findings of PBF-LB/M process on Tantalum and its alloys.

Reference	Material	Process	Acquired Results	Remarks
Livescu et al. [65]	Tantalum with 50/50 blend of TEKMAT™ Ta-25 and TEKMAT™ Ta-45	PBF-LB/M	Microstructure	The deposition parameters have a significant impact on the characteristics of the manufactured parts, particularly in terms of the desired crystallographic orientation, microstructure, and deposition porosity.
Sing et al. [66]	Ti-50Ta	PBF-LB/M	Microstructure Mechanical properties	There are several nuclei locations for grain formation observed along a single melt track of Ti-50T. Additionally, Ti-50T exhibits a combination of high strength and lower Young’s modulus when compared to commercially pure titanium and Ti6Al4V components.
Zhou et al. [64]	Pure Tantalum	PBF-LB/M	Densification Microstructure Mechanical properties	The quality of the part is significantly influenced by the phenomena known as the balling effect and Marangoni convection. The microhardness and tensile strength of pure Ta, as produced in this study, exhibited significant enhancements when compared to the powder metallurgy samples.
Thijs et al. [63]	Pure Tantalum	PBF-LB/M	Microstructure Crystallographic texture	Morphology and crystallographic texture result in considerable yield strength anisotropy. Crystallographic texture is the major source of the considerable differential in yield strength. The compression test results revealed good yield strengths. The yield strength of PBF-LB/M Ta is higher than the values provided for all the Ta other processes except cold works.
Sing et al. [67]	Ti-50Ta	PBF-LB/M	Influence of laser process parameters on the relative density and microhardness	The titanium-tantalum alloy generated by PBF-LB/M exhibits a relative density of 99.85 ± 0.18% and demonstrates favorable microhardness. This is achieved by utilizing a laser power of 360 W, a scan speed of 400 mm/s, a powder layer thickness of 0.05 mm, and a hatch spacing of 0.125 mm.
Wauthle et al. [68]	Pure Tantalum	PBF-LB/M	In vivo porous Ta, load- bearing bone defect model	Tantalum has remarkable osteoconductive characteristics, possesses a superior normalized fatigue strength, and enables greater plastic deformation owing to its elevated ductility.
Guo et al. [69]	Pure Tantalum	PBF-LB/M	Cytocompatibility in vitro and osseointegration capabilities in vivo	Ta scaffold group shows superior cell adhesion and proliferation results of human bone mesenchymal stem cells (hBMSCs) compared with the control porous Ti6Al4V group
Zhou et al. [70]	The influence of Tantalum content on phase change processes in the Ti-13Nb-13Zr alloy.	PBF-LB/M	The study investigates the association between phase transformations (namely β→ω and ω→α + β) and the elastic characteristics	The elastic modulus of the alloys exhibits an initial increase followed by a drop as the Ta content increases, primarily due to the subsequent variations in the quantity of the ω phase.
Zhang et al. [71]	Pure Tantalum	PBF-LB/M	Mechanical properties of lattice structures such as the imitation saddle surface (ISS) and the imitation arch bridge telescopic (IABT)	The ISS lattice exhibits the highest promise as a contender for bone implant applications.
Wang et al. [72]	Tantalum and Titanium	PBF-LB/M	Biomechanically suited porous Ta and Ti materials were fabricated, and a comparative analysis of their osteointegration and osteogenesis properties was conducted.	Porous scaffolds implanted in rabbit femur bone defects improved bone ingrowth and bone-implant fixation in vivo. In minor bone defect repair, porous Ta performs similarly to porous Ti implants. Overall, porous Ta appears promising for bone repair.
Huang et al. [73]	Ti-Ta with a wt% of 0, 10, 30 and 50%	PBF-LB/M	The investigation of the appropriateness of Ti-Ta alloys with varying Ta concentrations for biomedical applications.	Ti50Ta scaffolds produced biocompatibility comparable to Ti6Al4V and commercially pure titanium. This conclusion is drawn from the results acquired from cell culture experiments with the SAOS-2 human osteosarcoma cell line, which demonstrate similar biological outcomes and manufacturability.
Lian et al. [74]	Pure Tantalum	PBF-LB/M	Effect of Microstructure and Impurities on mechanical properties	The toughness of tantalum has been seen to exhibit a considerable increase as a result of a reduction in pore defects and oxygen-nitrogen impurities.
Song et al. [75]	Pure Tantalum	PBF-LB/M	The impact of scanning speed on the development of microstructure and mechanical properties	Research findings indicate that an increase in scanning speed results in the refinement of columnar grains in Ta. With a scanning speed of 800 mm/s, the sample exhibits exceptional mechanical properties, including a tensile strength of up to 706 MPa and a fracture elongation of up to 33.26%.
Fox et al. [46]	Titanium and Tantalum coating on a substrate of Co-28 Cr-6 Mo	PBF-LB/M	Comparison of interface interaction of Ti and Ta coating on the substrate.	Ta coatings exhibited significantly enhanced performance in terms of interface compatibility in comparison to titanium.

**Table 4:** Area porosity and volumetric energy density were adopted for pure Ta samples [65].

Parts	Area Porosity [%]	*E_d_* [J/mm^3^]
Sample cube 51	2.85	133.48
Sample cube 61	0.46	209.28
Sample cube 65	0.25	349.06
Sample cube 70	0.15	465.41
Sample cube 71	0.026	654.17
Sample cube 73	0.021	872.64
Sample cube 74	0.029	840.91
Sample cube 76	0.021	840.91
Sample cube 78	0.020	840.91
Sample plate 3	0.018	840.91

**Table 5:** Microhardness of pure Ta parts through various technologies [64].

Process	Vickers Microhardness	Reference
PBF-LB/M	425	Zhou et al. [64]
Casting	110	Balla et al. [84]
Powder metallurgy	120	Balla et al. [84]
Soft annealing	60–120	Cardonne et al. [85]
Cold working	102–200	Michaluk et al. [86]

**Table 6:** Mechanical properties of Ta-Ti alloys by PBF-LB/M with various Ta contents [67].

Alloy	Ultimate Tensile Strength (MPa)	Yield Strength (MPa)	Young’s Modulus (GPa)	Microhardness (HV)
Ti	641 ± 10	560 ± 13	115 ± 5	257 ± 7
Ti–6Ta	697 ± 2	595 ± 5	108 ± 4	241 ± 3
Ti–12Ta	783 ± 18	650 ± 7	99 ± 6	267 ± 10
Ti–18Ta	808 ± 17	668 ± 20	96 ± 3	296 ± 10
Ti–25Ta	1186 ± 14	1029 ± 8	89 ± 4	353 ± 11

## Data Availability

Not applicable.
